# The Central Texas Medical Society

**Published:** 1892-10

**Authors:** 


THE CENTRAL TEXAS MEDICAL SOCIETY and the Austin District Medical Society have formed a mutual admiration league, and are swapping visits. It will be remembered that the Central Texas Society accepted an invitation from the A. D. S., and met with them in joint session last June, holding a very interesting and profitable meeting, a full report of which, with the discussions, was published in DANIEL'S TEXAS MEDICAL JOURNAL. Quinine was the "bone of contention" then, and the bone was extensively "gnawed." The Centrals reciprocated the courtesy by sending the A. D. S. an invitation to meet with them at Waco on the 11th of October (inst.), which invitation was accepted; some twenty of the latter responding. "Circumstances over which we had no control" prevented the representative of the JOURNAL from being present, much to his regret, and consequently we are unable to treat our readers to a write up of the meeting. The Waco *Day* gave but an outline of the meeting, about as interesting as a last year's almanac; it consisted mostly of a list of names of those present, most of them spelled wrong at that; for instance, the celebrated Dr. Hadra was put down as

"Dr. Hades." (Such is fame!) If the doctor should visit Waco again "Col." Davis, the Waco *Day* or (break o' day) man had better go hunting, or he will catch Hades. Dr. Bennett attended, but the JOURNAL has not been enabled to extract enough from him to weave a report on; about all he could tells us was that there was a banquet. The *Day* did not even report the banquet speeches, and of course all the speakers were down on "Col." Davis next day.

Well, about the quinine, we did hear that they took up the subject where they left off at the Austin meeting, and like Jones' fight, they "had it around and around," but unlike Jones' fight, we don't know who "at last, hollered." "Dr. Hades" remarked that if the patient be a strong, healthy man of a robust constitution he might survive the doses which Dr. Blank recommends, while Dr. Sears is on record for owning up to an ounce a week in his own case. It will be remembered he had lymphangitis or something, afterwards, and he wanted to know of the A. D. S., if it could have been milk-leg. Whether quinine was finally disposed of, or whether it will bob up at the next reunion, remains to be seen; we are staking ours on its bobbing.

Dr. Blailock, President of the Wacos, and Christian, of the A. D. S., each read a paper, which, we are told were very good. Readers will please accept our apologies for being unable to give a good report of that important occasion.

